# Diagnostic Challenges in Rhino-Orbito-Cerebral Mucormycosis With Diabetes Mellitus, Hypertensive Emergency, and Excess Alcohol Consumption

**DOI:** 10.7759/cureus.74663

**Published:** 2024-11-28

**Authors:** Moaz Mohsin, Oluwaseyi Owasoyo, Hafiz Muhammad Zahid Rahim, Uzma Rahim, Asma Rahim, Swaroopa Kinnera, Kashish Keswani Sunil, Abrar A Awan, Ayesha Hassan, Shahzaib Munir

**Affiliations:** 1 Acute Medicine, Royal Stoke University Hospital, Stoke-on-Trent, GBR; 2 Stroke, Queens Hospital Center, Romford, GBR; 3 Psychiatry, Withybush General Hospital, Haverfordwest, GBR

**Keywords:** alcohol dependence, diabetes mellitus, diabetic ketoacidosis (dka), hyperglycemia, hypertension, hypertensive emergency, mucormycosis, retinal artery occulusion, rhinocerebral mucormycosis, rhino-orbito-cerebral mucormycosis

## Abstract

A 72-year-old male with a history of excessive alcohol intake (35+ units of alcohol daily) presented to the emergency department with bilateral vision loss, periorbital swelling, headache, and sinus congestion with bloody nasal discharge. He was newly diagnosed with diabetes mellitus and presented with severe diabetic ketoacidosis and a hypertensive emergency (blood pressure of 240/90 mmHg). Despite initial normal brain and orbital imaging, the clinical presentation was complicated by multiple life-threatening conditions and a prior immunocompetent status, thereby delaying the early suspicion of mucormycosis. This case underlines the diagnostic challenges in managing mucormycosis in a previously immunocompetent patient with overlapping critical conditions.

## Introduction

Mucormycosis is a life-threatening opportunistic fungal infection caused by several fungi belonging to the phylum Glomeromycota [1]. These fungi of the order Mucorales including Rhizopus, Lichtheimia, Apophysomyces, Mucor, Absidia, and Rhizomucor, which are the most common perpetuators of the infection in humans, are usually found as ubiquitous saprophytes on decaying soil and plant substrates [1-3]. 

Mucormycosis is a rare infection with an incidence rate of 0.09 per 100,000 each year in the United Kingdom [4]. It primarily affects individuals who are immunocompromised, on long-term steroid therapy, have malignancies, are acidotic, or have uncontrolled diabetes mellitus (DM) complicated by diabetic ketoacidosis (DKA); with diabetes being the most common risk factor [5]. Infection spreads as spores via inhalation, cuts, wounds, or ingestion of contaminated foods [6], with presenting symptoms dependent on the type and distribution of the infection [1,6]. The most common forms are rhino-orbito-cerebral (ROC), pulmonary, cutaneous, gastrointestinal, disseminated, and isolated renal mucormycosis [7,8]. 

Rhino-orbito-cerebral mucormycosis (ROCM) is the most common subtype of mucormycosis, accounting for approximately 40-50% of cases with about 60-80% of cases occurring in patients with DKA [7,8]. With non-specific presenting symptoms, the clinical suspicion and eventual management of this rare and potentially fatal infection pose a great diagnostic challenge if not diagnosed early [9]. 

This report not only highlights the challenge of diagnosing mucormycosis early, but also the difficulties faced when encountered with multiple other life-threatening presentations.

## Case presentation

A 72-year-old male presented to the emergency department with a 24-hour history of sudden bilateral vision loss, preceded by left-sided facial pain, headache, peri-orbital swelling, blocked sinuses, bloody nasal discharge, nausea, vomiting, and abdominal pain. 

His medical background included a significant history of alcohol use (35+ daily units). Though he had no known history of diabetes prior to presentation, lab investigations revealed severe metabolic acidosis and ketosis, with blood glucose levels of 24 mmol/L, ketones of 6 mmol/L, and HbA1c levels of 135 mmol/mol, confirming a diagnosis of DM with severe DKA. The patient's laboratory investigations are included in Table 1. 

**Table 1:** Biochemical and Haematological investigations at presentation

Parameter	Value	Reference range
pH	6.93	7.35-7.45
Bicarbonate	8.10	22-32 mmol/L
Glucose	24.0	3.9-5.6 mmol/L
Ketones	6	<0.6 mmol/L
Biochemistry		
Serum sodium	134	133-146 mmol/L
Serum potassium	4.7	3.5-5.3 mmol/L
Urea	8.4	2.5-7.8 mmol/L
Creatinine	69	59-104 µmol/L
C-Reactive Protein	153	0-5 mg/L
ESR	19	2-18 mm/hr
Haematology		
Haemoglobin	164	130-180 g/L
White Cell Count	11.00	4.0-11.0 x10^9^/L
Platelets	252	150-450 x10^9^/L
Neutrophils	9.57	2.0-7.5 x10^9^/L
Lymphocytes	0.45	1.5-4.0 x10^9^/L
Monocytes	0.80	0.20-0.80 x10^9^/L
Eosinophils	0.01	0.04-0.40 x10^9^/L

On examination, he was also found to be hypertensive, with a blood pressure of 240/90 mmHg, marked bilateral periorbital swelling, complete vision loss, and complete ophthalmoplegia. Both pupils were fixed and non-reactive. His Glasgow Coma Scale (GCS) was 15 (E4[blind], V5, M6). Fundoscopy revealed pale retinas with cherry-red spots, raising concern for bilateral ophthalmic artery occlusion. Rhinoscopy showed a black nasal septum initially attributed to dry blood from a week-long history of epistaxis. 

The initial management focused on addressing hypertensive encephalopathy and correcting metabolic abnormalities. The patient was initiated on fixed-rate insulin infusion according to the local DKA protocol and glyceryl trinitrate (GTN) infusion to manage hypertension and keep the systolic blood pressure below 160 mmHg. The insulin and GTN were effective in managing the DKA and hypertensive emergency. At this point in treatment, a broad range of differential diagnoses was considered, including methanol poisoning, hypertensive crisis, bilateral central retinal artery occlusion (CRAO), giant cell arteritis (GCA), and posterior reversible encephalopathy syndrome (PRES). Given the fundoscopy findings and the complete loss of vision from both eyes with a marginal rise in the erythrocyte sedimentation rate, GCA was high on the list of differentials. A decision was reached to use intravenous pulse corticosteroids to recover the vision. Once the hyperglycemia had settled, the patient was empirically treated with intravenous methylprednisolone (1 gram every eight hours) and intravenous antibiotics while further diagnostic efforts were underway. 

**Figure 1:**
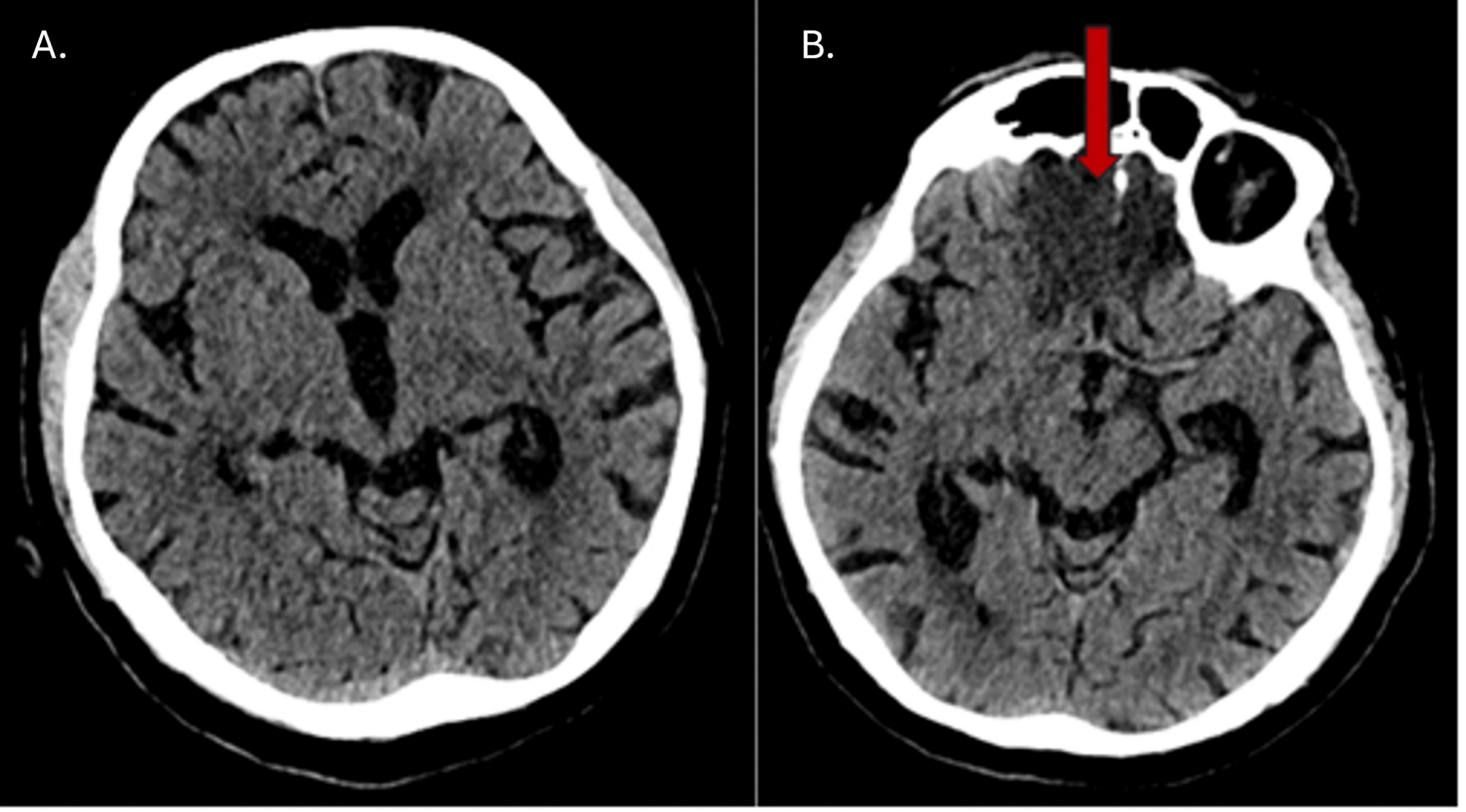
A: Normal CT Head on the day of admission. B: Hypoattenuation in the frontal lobes (red arrow) with patent anterior cerebral arteries. CT was obtained on the third day of admission.

A computed tomography (CT) head scan initially showed no abnormalities (Figure 1A). Magnetic resonance imaging (MRI) of the head and orbits and with MR angiogram revealed a high diffusion signal restriction at the base of the right frontal lobe without T2 FLAIR enhancement, a finding initially attributed to DKA. No abnormality of the orbits, optic nerve, brain, or sinuses was detected on MRI. 

By day three, the patient’s clinical status deteriorated, with necrosis developing at the medial canthus of the right eye and a decrease in GCS score to 9 (E2, V3, M4). The patient became more agitated and developed hypertensive emergency with encephalopathy once again with a blood pressure of 235/137 mmHg. Consequently, he was restarted on a GTN infusion, and repeat imaging was obtained under sedation. He was transferred and further managed in the intensive treatment unit (ITU). Repeat imaging showed new bilateral inferior frontal lobe infarcts, while the anterior cerebral arteries remained patent (Figure 1B). The optic nerve appeared normal, but thickening of the mucosa in all sinuses was now evident. With these updated findings, mucormycosis was considered as a potential diagnosis, given the progression of orbital and cerebral involvement. 

In response, intravenous methylprednisolone was discontinued, and he was immediately started on a high-dose intravenous infusion of Liposomal amphotericin B 10mg/kg daily. Urgent functional endoscopic sinus surgery (FESS) was performed to obtain tissue biopsies, which revealed extensive necrosis throughout the nasal cavity. Additional tests, including HIV screening and blood film microscopy, showed no signs of immunocompromise. Microscopic analysis of the biopsies demonstrated features of fungal infection with tissue and angioinvasion as well as widespread necrosis. Culture results showed pauci-septate filamentous fungus confirmed to be *Rhizopus Arrhizus*, consistent with mucormycosis. 

Given the extensive orbital and cerebral involvement, the prognosis was poor. Aggressive surgical options, including potential enucleation, were considered but deemed unlikely to improve the patient's quality of life due to extensive tissue destruction and the high risk of further CNS involvement. By this point, the patient was intubated and ventilated with progression of necrosis despite aggressive medical treatments in the ITU setting. Given the impractical surgical and failing medical interventions, after discussion with a multidisciplinary team and the patient’s family, a decision was reached to withdraw life-sustaining treatments and the patient passed away thereafter. 

## Discussion

Mucormycosis, which was first recognized by J.E. Gregory in 1943, is an acutely rapidly progressive opportunistic, aggressive and fatal disease caused by angiotrophic fungi of the Mucorales order [2,3,7,9,10].  ROCM is the most encountered presentation in clinical practice, with the *Rhizopus oryzae* (Arrhizus) species being the commonest causative agent [2,9]. 

Diabetes mellitus, particularly uncontrolled diabetes and DKA, is purported to be the most important risk factor for the disease and is implicated in about 60-80% of cases worldwide, with poorly controlled blood sugars at presentation being linked with adverse clinical outcomes [2,3,7]. Other risk factors include steroid/glucocorticoid therapy, malignancies such as lymphomas and leukaemias, retroviral disease, renal failure, long-term immunosuppressive therapy, and malnutrition among others [2,8]. 

The infection begins at the nasal turbinates, which is usually the first site of infection [2], with inhalation of airborne Mucorales spores germinating into hyphae, causing acute sinusitis and subsequent colonization of the nose, oral mucous membranes, oropharynx and paranasal sinuses with further rapid progression to the palate, orbit, and brain culminating in severe tissue ischemia and necrosis secondary to the fungi’s angio-invasive nature. This eventually results in rapid vascular and neuronal compromise, including thrombosis, tissue necrosis, ulceration, infarction, eschar formation, bone and meningeal involvement, and infection along the ethmoid and sphenoid sinuses, with subsequent extensive thrombosis of the cavernous sinus, jugular veins, and carotid artery [2,8]. The *Rhizopus* species can thrive incredibly in a hyperglycaemic state like DKA, owing to its ketone reductase system, coupled with impaired phagocytosis caused by hyperglycaemia. Therefore, steroid therapy, which ultimately aggravates hyperglycaemia, is well known for impairing phagocytosis [8]. 

The clinical manifestations of this disease are often initially non-specific, including headache, fever, nasal discharge with or without epistaxis, nasal necrosis, sinusitis, and facial pain and/or numbness [2]. Orbital involvement typically includes ptosis, proptosis, diplopia, ophthalmoplegia, corneal oedema, optic neuritis, CRAO, and visual loss [2,7,9]. In severe cases with involvement of the brain, patients may also present with altered consciousness, coma, unsteady gait, multiple cranial nerve palsies and even seizures as well as cavernous sinus thrombosis (CST), which is a marker of poor prognosis [2,9]. 

The varied symptomatology of this disease in addition to rapid disease progression poses a diagnostic challenge. A high index of suspicion is therefore vital to early diagnosis and improved clinical outcomes [2]. While CT scanning is useful in assessing the extent and advancement of disease including soft tissue involvement of the sinuses and the brain, as well as mucosal and bony erosions/necrosis, MRI is more useful for highlighting vascular invasion as well as intracranial and perineural extension or involvement [2,7]. Nevertheless, imaging is not the gold standard method of diagnosis and should not be relied upon as normal imaging does not sufficiently rule out the presence of the infection as was evident from this case. The mainstay of diagnosis, however, involves direct histological/cytopathological visualization of characteristic ribbon-shaped septate or pauci-septate mycelial filaments from infected tissue specimens with demonstrable surrounding necrosis, thrombosis, and haemorrhage [2,9]. 

Managing this life-threatening disease first involves prompt and early recognition and diagnosis, as it is classed as a surgical and medical emergency [2]. Treatment involves a combination of antifungal therapy with intravenous amphotericin (liposomal) 5-10 mg/kg/day for several weeks followed by oral step-down either to posaconazole or isavuconazole, coupled with extensive surgical debridement and resection of necrotic tissue to enhance penetration of antifungal agents, as well as the correction or reversal of predisposing factors and immunosuppressive states, including hyperglycaemia, ketoacidosis, and neutropenia, among others [2,9,11]. 

Unfortunately, the prognosis for this rare disease is very poor and is inherently affected by several factors. Firstly, better outcomes are documented in cases with only sinus involvement, with a reported 50 to 80% survival documented in patients where there is no brain involvement. This is in contrast to only 20% survival documented in patients presenting with brain involvement [2]. Furthermore, the presence of visual symptoms at presentation, delay between presentation and diagnosis/ initiation of treatment, hyperglycaemia, and neurological involvement are all associated with poor patient outcomes [2,7,9]. 

This case highlights the diagnostic and therapeutic challenges associated with mucormycosis, particularly in patients with multiple co-existing emergencies. Our patient presented with uncontrolled hypertension and excessive drinking which are not a commonly presenting feature alongside mucormycosis. Notably, the visual symptoms were initially thought to be secondary to DKA, hypertension and excess ethanol consumption. Normal imaging on admission further delayed the suspicion of mucormycosis. The addition of intravenous corticosteroids for the treatment of suspected GCA also added to the risk factors for fatal disease progression, along with uncontrolled diabetes and metabolic acidosis that further encouraged the rapid spread of the infection. 

Despite early initiation of antifungal therapy and management of risk factors, the infection progressed rapidly, as there were unfortunately multiple odds that challenged and posed difficulties for a positive outcome. This difficult case underscores the importance of early suspicion, prompt surgical intervention, and aggressive management in improving outcomes in mucormycosis infections. However, even with treatment, the prognosis for mucormycosis remains quite bleak. 

## Conclusions

Mucormycosis is a rare, rapidly progressing fungal infection with high mortality and low incidence rates. This case highlights the need for heightened awareness and a high incidence of suspicion to aid early diagnosis of mucormycosis infections, to ensure favourable patient outcomes, and avoid rapid disease progression. Over-reliance on imaging modalities should be avoided as positive image findings can be a late feature of disease progression. Better awareness of this infection among clinicians may lead to early suspicion, recognition, and intervention to avoid disease progression and achieve lower mortality with improved patient quality of life. 
